# Evaluation of Pneumococcal Serotyping of Nasopharyngeal-Carriage Isolates by Latex Agglutination, Whole-Genome Sequencing (PneumoCaT), and DNA Microarray in a High-Pneumococcal-Carriage-Prevalence Population in Malawi

**DOI:** 10.1128/JCM.02103-20

**Published:** 2020-12-17

**Authors:** Todd D. Swarthout, Andrea Gori, Naor Bar-Zeev, Arox W. Kamng’ona, Thandie S. Mwalukomo, Farouck Bonomali, Roseline Nyirenda, Comfort Brown, Jacquline Msefula, Dean Everett, Charles Mwansambo, Katherine Gould, Jason Hinds, Robert S. Heyderman, Neil French

**Affiliations:** a Malawi-Liverpool-Wellcome Trust Clinical Research Programme, Blantyre, Malawi; b NIHR Global Health Research Unit on Mucosal Pathogens, Division of Infection and Immunity, University College London, London, United Kingdom; c International Vaccine Access Center, Department of International Health, Johns Hopkins University, Baltimore, Maryland, USA; d Department of Biomedical Sciences, College of Medicine, University of Malawi, Blantyre, Malawi; e Department of Medicine, College of Medicine, University of Malawi, Blantyre, Malawi; f The Queens Medical Research Institute, University of Edinburgh, Edinburgh, Scotland; g Ministry of Health, Lilongwe, Malawi; h Institute for Infection and Immunity, St. George’s, University of London, London, United Kingdom; i BUGS Bioscience, London Bioscience Innovation Centre, London, United Kingdom; j Centre for Global Vaccine Research, Institute of Infection and Global Health, University of Liverpool, Liverpool, United Kingdom; University of Iowa College of Medicine

**Keywords:** *Streptococcus pneumoniae*, serotyping, latex agglutination, microarray, whole-genome sequencing, methodology, Africa

## Abstract

Accurate assessment of the serotype distribution associated with pneumococcal colonization and disease is essential for evaluating and formulating pneumococcal vaccines and for informing vaccine policy. For this reason, we evaluated the concordance between pneumococcal serotyping results by latex agglutination, whole-genome sequencing (WGS) with PneumoCaT, and DNA microarray for samples from community carriage surveillance in Blantyre, Malawi. Nasopharyngeal swabs were collected according to WHO recommendations between 2015 and 2017 by using stratified random sampling among study populations. Participants included healthy children 3 to 6 years old (vaccinated with the 13-valent pneumococcal conjugate vaccine [PCV13] as part of the Expanded Program on Immunization [EPI]), healthy children 5 to 10 years old (age-ineligible for PCV13), and HIV-infected adults (18 to 40 years old) on antiretroviral therapy (ART). For phenotypic serotyping, we used a 13-valent latex kit (Statens Serum Institut [SSI], Denmark). For genomic serotyping, we applied the PneumoCaT pipeline to whole-genome sequence libraries. For molecular serotyping by microarray, we used the BUGS Bioscience Senti-SP microarray. A total of 1,347 samples were analyzed. Concordance was 90.7% (95% confidence interval [CI], 89.0 to 92.2%) between latex agglutination and PneumoCaT, 95.2% (95% CI, 93.9 to 96.3%) between latex agglutination and the microarray, and 96.6% (95% CI, 95.5 to 97.5%) between the microarray and PneumoCaT. By detecting additional vaccine serotype (VT) pneumococci carried at low relative abundances (median, 8%), the microarray increased VT detection by 31.5% over that by latex serotyping. To conclude, all three serotyping methods were highly concordant in identifying dominant serotypes. Latex serotyping is accurate in identifying vaccine serotypes and requires the least expertise and resources for field implementation and analysis. However, WGS, which adds population structure, and microarray, which adds multiple-serotype carriage, should be considered at regional reference laboratories for investigating the importance of vaccine serotypes at low relative abundances in transmission and disease.

## Abstract (precis)

Accurate assessment of the serotype distribution associated with pneumococcal colonization and disease is essential for evaluating and formulating pneumococcal vaccines and for informing vaccine policy. For this reason, we evaluated the concordance between pneumococcal serotyping results by latex agglutination, whole-genome sequencing (WGS) with PneumoCaT, and DNA microarray for samples from community carriage surveillance in Blantyre, Malawi. Nasopharyngeal swabs were collected according to WHO recommendations between 2015 and 2017 by using stratified random sampling among study populations.

## INTRODUCTION

Streptococcus pneumoniae colonizes the nasopharynx of healthy individuals. Although carriage is usually asymptomatic, nasopharyngeal (NP) colonization is a prerequisite for diseases including otitis media, sinusitis, pneumonia, bacteremia, and meningitis (1). The pneumococcus is estimated to be responsible for >318,000 (uncertainty ratio [UR], 207,000 to 395,000) deaths every year in children aged 1 to 59 months, with the highest mortality burden among African children (2). Evidence also shows that HIV-infected children and adults are at significantly higher risk of invasive pneumococcal disease (IPD) than their non-HIV-infected counterparts (3, 4).

Current multivalent pneumococcal conjugate vaccines (PCV) target subsets of the 100 capsular serotypes known to be expressed by the pneumococcus. PCV reduce nasopharyngeal carriage of the subset of pneumococcal serotypes they contain, known as vaccine serotypes (VT). With reduced carriage among the vaccinated, there is then a reduced risk of VT-IPD (direct protection) and reduced transmission, leading to a reduced risk of VT-IPD among those not vaccinated with PCV (indirect protection). However, nonvaccine serotypes (NVT) have the potential to fill the ecological niche, becoming more common in carriage and disease (5–7). This phenomenon, known as serotype replacement, may be more pronounced in low-income settings because of the higher prevalence, density, and diversity of pneumococcal carriage, and it represents a considerable risk to the global pneumococcal immunization strategy (8). Serotype distribution differs among continents as well as among individual countries (9). Given these differences, accurate assessment of the serotype distribution associated with both pneumococcal colonization and pneumococcal disease is needed in the evaluation, formulation, and delivery of pneumococcal vaccines.

A pneumococcal serotyping method suitable for use in robust carriage and surveillance studies should therefore, at minimum, be accurate in its serotype assignment, particularly in relation to VT. Additional desirable parameters include detection of most or all serotypes, the ability to detect multiple serotypes in carriage (common in high-burden settings [10, 11]), in-depth information on genotype, suitability for scale-up to large projects, and practicality for resource-poor settings. Unfortunately, work in resource-poor settings too often limits the number of these parameters that can be achieved.

The gold standard serotyping method, the Quellung reaction, was developed in the early 1900s and is performed by testing colonies with a set of type-specific antisera (12). Bacteria are observed by microscopy, and the serotype is defined by observing apparent capsular swelling in reaction to type-specific antisera. This method is laborious, requires frequent use to maintain skills, and requires a complete set of type-specific antisera; therefore, it is performed mainly by reference laboratories. The PneuCarriage Project, a large multicenter study, was established with the aim of identifying the best pneumococcal serotyping methods for carriage studies (13). The Project identified microarray with a culture amplification step as the top-performing method. While robust and systematic, their decision algorithm did not take into account parameters such as cost, skill level, resources needed for assay implementation and maintenance, output processing, and interpretation.

Here, we describe, in the context of an ongoing field-based study (14), the levels of concordance among three methods commonly used during ongoing routine pneumococcal surveillance activities in our work: latex agglutination, microarray, and serotyping by sequencing. We also address parameters that researchers and policy makers can consider when deciding which assay to implement in their local settings.

## MATERIALS AND METHODS

### Study setting.

Blantyre is located in southern Malawi with an urban population of approximately 1.3 million.

### Study population and recruitment.

Samples were collected as part of a larger 3.5-year pneumococcal carriage surveillance project, as described elsewhere (14). In brief, this was a prospective rolling cross-sectional observational study using stratified random sampling to measure nasopharyngeal pneumococcal carriage in Blantyre, Malawi. The samples used in this analysis were collected during the first 2 years of twice-annual cross-sectional surveys, from June 2015 to April 2017. Recruitment included three groups: (i) healthy children 3 to 6 years old who received the 13-valent pneumococcal conjugate vaccine (PCV13) as part of routine EPI [Expanded Program on Immunization] activities, (ii) healthy children 5 to 10 years old who were age-ineligible to receive PCV13 as part of EPI, and (iii) HIV-infected adults (18 to 40 years old) on antiretroviral therapy (ART).

### Sample selection.

**FIG 1:**
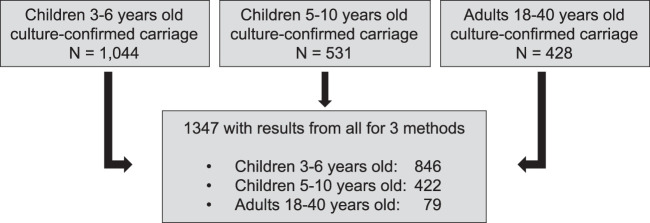
Sample selection for analysis. Samples were collected during four rolling cross-sectional surveys from June 2015 to April 2017. Of the total nasopharyngeal swab samples collected, 1,347 had results available from the three assays under review. Selection for the microarray and PneumoCaT was done independently of available latex serotyping data.

For analyses of concordance among the three methods, all samples were included that had serotyping results available from each of the three methods (latex agglutination, microarray, serotyping by sequencing). From the total nasopharyngeal swab (NPS) samples collected during the larger surveillance project (including 1,044 from children 3 to 6 years old [PCV vaccinated], 531 from children 5 to 10 years old [not PCV vaccinated, age-ineligible], and 428 from HIV-infected adults on ART), 1,347 samples were culture confirmed for S. pneumoniae and also had results available from the microarray and serotyping by sequencing. The final concordance analysis included 846 children 3 to 6 years old (PCV13 vaccinated), 422 children 5 to 10 years old (age-ineligible for PCV13 vaccination), and 79 adults (HIV infected and not PCV13 vaccinated) (Fig. 1). Samples for the microarray and serotyping by sequencing were selected independently in a manner blind to latex serotyping data.

### Nasopharyngeal swab collection.

**FIG 2:**
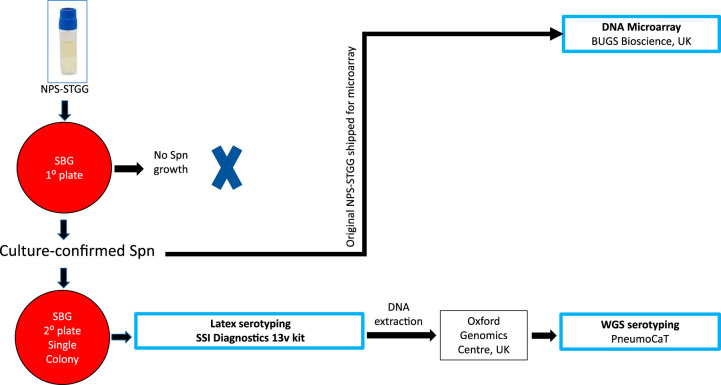
Laboratory procedures. Nasopharyngeal swabs were inoculated into STGG medium and subsequently plated on a growth agar of sheep blood and gentamicin. Bacterial growth (from single-colony picks) from samples culture confirmed for Streptococcus pneumoniae was used for latex serotyping. The remaining pure-growth isolates, retained at –80°C in sterile STGG, were later grown for DNA extraction and WGS. Aliquots of original samples (NPS-STGG) that were culture confirmed for Streptococcus pneumoniae were assessed by microarray. NPS, nasopharyngeal swabs; STGG, skim milk-tryptone-glucose-glycerol; WGS, whole-genome sequencing; Spn, Streptococcus pneumoniae; SBG, sheep blood and gentamicin; SSI, Statens Serum Institut; 13v, 13-valent; NPS-STGG, NPS inoculated into STGG.

The collection of NP swabs has been described elsewhere (14). In brief, an NP swab sample was collected from each participant by using a nylon flocked swab (FLOQSwabs; Copan Diagnostics, Murrieta, CA, USA), immediately placed in 1.5 ml skim milk-tryptone-glucose-glycerol (STGG) medium, and processed at the Malawi–Liverpool–Wellcome Trust (MLW) laboratory in Blantyre, Malawi, according to WHO recommendations (15). Samples were frozen on the same day at −80°C (Fig. 2).

### NPS culture for pneumococcal screening and serotyping.

A 30-μl portion of NPS–STGG was plated onto a sterile sheep blood-gentamicin (SBG; 7% sheep blood agar [SBA], 5 μl gentamicin/ml) agar plate (primary plate) and incubated overnight at 37°C under ∼5% CO_2_. Plates showing no S. pneumoniae growth were incubated overnight a second time before being reported as negative. S. pneumoniae was identified by colony morphology and optochin disc (Oxoid, Basingstoke, UK) susceptibility. The bile solubility test was used on isolates with no or intermediate (zone diameter, <14 mm) optochin susceptibility. A single colony of confirmed pneumococcus was selected and grown on a sterile SBG plate (secondary plate) by following the same process as that for the primary plate (Fig. 2).

### Latex serotyping.

Pneumococcal growth from secondary plates was used for serotyping by latex agglutination (ImmuLex 7-10-13-valent Pneumotest; Statens Serum Institute, Denmark) according to manufacturer guidelines. Using a reaction card and a sterile inoculation loop, a small sweep of an overnight bacterial culture was mixed with saline and a series of individual Pneumotest-Latex reagents in suspension. The card was rocked manually and observed for agglutination. A Pneumotest-Latex chessboard was used to determine which serotype was associated with the observed set of agglutination reactions. The kit allows for differential identification of each PCV13 VT (serotypes 1, 3, 4, 5, 6A, 6B, 7F, 9V, 14, 18C, 19A, 19F, and 23F). Other than for a limited number of serogroups (serogroups 6, 7, 9, 18, 19, and 23) for which the kit provides serogroup differentiation, there is no further differential identification of NVT serogroups or serotypes. NVT and nontypeable isolates were reported as NVT. Samples were batch-tested on a weekly basis, with technicians blinded to the sample source. After serotyping was complete, the remaining growth from each secondary plate was archived at –80°C in sterile STGG. For a more detailed description of latex serotyping, see Text S1 in the supplemental material.

### Molecular serotyping by microarray.

For samples with culture-confirmed pneumococcal carriage, the original inoculated STGG was thawed and vortexed. Aliquots of 100 μl were shipped in 1.8-ml cryovials to BUGS Bioscience Ltd. (London, United Kingdom) on dry ice (Fig. 2). The remaining steps for microarray serotyping (including sample processing, culturing, DNA extraction, microarray, and analysis) were completed entirely by BUGS Bioscience (16, 17). Final microarray results were retrieved by the study team from the BUGS Bioscience Web-based SentiNET platform and imported into STATA 13.1 (StataCorp, College Station, TX, USA) for analysis. Refer to Text S1 for a more detailed description of microarray serotyping.

### DNA extraction and WGS.

Archived secondary-growth isolates were used to develop sequence libraries for serotyping by sequencing. To optimize total retrieved DNA, 30 μl of thawed isolate-STGG was incubated overnight in 6 ml THY (Todd-Hewitt broth plus yeast) enrichment culture. DNA was extracted from the overnight culture using the Qiagen QIAamp DNA minikit according to manufacturer guidelines for bacterial DNA. Quality control (QC) measures, as required by the guidelines of the sequencing institution, included DNA quantification (Qubit; Thermo Fisher Scientific, MA, USA) for all DNA samples and gel electrophoresis imaging on 0.7% agarose to assess DNA integrity. After quantity and quality requirements were met, 100 μl of extracted DNA was aliquoted into skirted 96-well microwell plates and stored at –80°C until it was shipped on dry ice to the Oxford Genomics Centre (University of Oxford, Oxford, United Kingdom) for sequencing. Whole-genome sequencing (WGS) was performed at the Oxford Genomics Centre on a HiSeq 4000 platform (Illumina), with paired-end libraries and a read length of 150 bp.

### Serotyping by sequencing.

WGS data were retrieved by the study team from a Web-based FTP link. Serotypes were inferred from the isolates’ genome sequences using the PneumoCaT software pipeline, an open-source bioinformatic tool (18). PneumoCaT requires raw sequencing reads for each isolate; these were trimmed and cleaned. Reads were trimmed of the Illumina adapters and cleaned of low-quality ends using Trimmomatic (version 0.38; available at http://www.usadellab.org/cms/?page=trimmomatic). The minimum read length after trimming was 80 bp, and the minimum average quality for a sliding window of 4 nucleotides was 15. A subset of 700,000 reads per end (1.4 million total) was used for any subsequent analysis. XML result files were parsed with *ad hoc* bash scripts in order to extract and tabulate the serotyping result for each isolate. PneumoCaT was installed and used on a Linux machine at the MRC Cloud Infrastructure for Microbial Bioinformatics (CLIMB [https://www.climb.ac.uk/]). Each serotype identification required an average of 5 to 8 min. Refer to Text S1 for a more detailed description of serotyping by sequencing.

### Definitions.

Concordance was calculated with all samples aggregated and according to the level of discrimination provided by the method. Concordance is reported using two criteria, based on (i) whether both assays reported NVT or both reported VT (VT/NVT criterion) and (ii) whether the final serotype reported by each assay was equivalent to the serotype reported by the other (serotype-specific criterion).

### (i) Concordance between latex agglutination and serotyping by sequencing (PneumoCaT).

Apart from a limited number of serogroups (serogroups 6, 7, 9, 18, 19, and 23) for which the latex kit provides differentiation, there is no further differential identification of NVT serogroups to serotype. NVT and nontypeable isolates were reported as NVT. Concordance at the serotype level (serotype-specific criterion) was reported only if latex serotyping reported VT carriage. If latex serotyping reported NVT, any NVT reported by PneumoCaT was considered concordant. For example, 23F reported by both latex and PneumoCaT was considered concordant, as were NVT and 15B. However, 19F and 19A were considered discordant, as were NVT and 6B.

### (ii) Concordance between latex agglutination and the microarray.

Concordance at the serotype level (serotype-specific criterion) was reported only if latex agglutination reported VT carriage. If latex serotyping reported NVT, any NVT reported by the microarray was considered concordant. Because the microarray reports multiple-serotype carriage, 23F reported by latex and 23F plus 34 reported by the microarray were considered concordant, as were NVT and 18C plus 33D. However, 19F and 33D plus 19A were considered discordant, as were NVT and 3 plus 7F.

Note that for the microarray, some closely related serotypes are reported as a group, with the final individual serotype call in brackets (e.g., 6A/B [6B]). In this case, results were analyzed using the individual serotype call. For example, if the microarray reported 6A/B [6B], this was considered discordant with a 6A latex agglutination result and concordant with a 6B latex agglutination result. For simplicity of analysis, if a method did not claim to detect a serotype but the sample contained that serotype, this result was deemed discordant. For example, if the microarray detected both serotypes 23F and 19A but latex agglutination detected only serotype 3, this result was considered discordant.

### (iii) Concordance between microarray and serotyping by sequencing (PneumoCat).

The microarray and PneumoCat both differentiate VT and NVT to serotype level, allowing concordance to be calculated according to the serotype-specific criterion for both VT and NVT S. pneumoniae.

### Statistical analysis.

The formula for the percentage of increase in VT prevalence was as follows: ([VT prevalence using latex agglutination – VT prevalence using microarray]/[VT prevalence using latex]) × 100%. Confidence intervals are binomial exact. Statistical significance was inferred from a two-sided test (*P* < 0.05). Participant data collection was completed using Open Data Kit (ODK) Collect open-source software (version 1.24.0). Statistical analyses were completed using Stata 13.1 (StataCorp, College Station, TX, USA).

### Ethics considerations.

The study protocol was approved by the College of Medicine Research and Ethics Committee, University of Malawi (P.02/15/1677), and the Liverpool School of Tropical Medicine Research Ethics Committee (14.056). Adult participants and parents/guardians of child participants provided written informed consent; children 8 years old and older provided informed assent. This included consent for publication.

### Data availability.

The data supporting the findings of this study have been deposited in the Figshare repository (26).

## RESULTS

**FIG 3:**
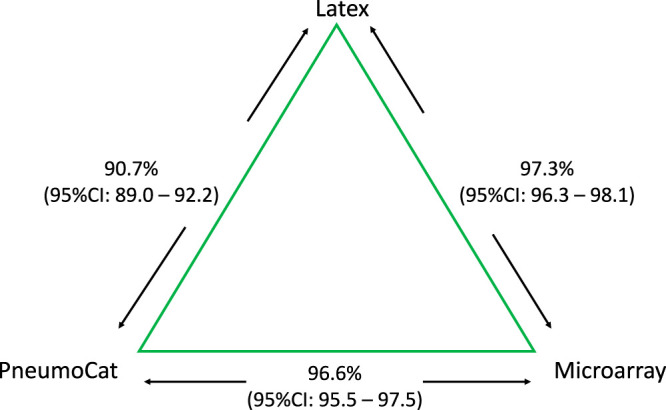
Concordance between assays. Concordance between two assays was defined as both assays identifying pneumococcal carriage as VT or as NVT. Latex agglutination and PneumoCaT reported one result per sample, both using the same pure-growth culture. The microarray, using an aliquot of the original NPS-STGG, differentiated individual serotypes in multiple-serotype carriage, when present. In comparing the three assays, concordance was based on serotype if latex serotyping reported VT carriage. If latex serotyping reported NVT carriage, this was considered concordant with any NVT reported by PneumoCaT or the microarray, as long as PneumoCaT and the microarray reported the same NVT.

Pneumococcal carriage prevalence results from the larger surveillance project have been reported elsewhere (14). In a comparison of latex agglutination and PneumoCat, the adjusted concordance of correctly identifying pneumococcal carriage as VT or NVT was 90.7% (1,216/1,341) (95% confidence interval [CI], 89.0 to 92.2%) (Fig. 3). Based on the serotype-specific criterion, concordance between latex agglutination and PneumoCaT was 87.5% (1,174/1,341) (95% CI, 85.7 to 89.3%). In a comparison of latex agglutination and the microarray, the concordance based on correctly identifying pneumococcal carriage as VT or NVT was 97.3% (1,311/1,347) (95% CI, 96.3 to 98.1%). Based on a serotype-specific criterion, the concordance was 95.2% (1,282/1,347) (95% CI, 93.9 to 96.3%). In a comparison of the microarray and PneumoCaT, concordance based on correctly identifying pneumococcal carriage as VT or NVT was 96.6% (1,295/1,341) (95% CI, 95.5 to 97.5%). Based on a serotype-specific criterion, the concordance was 82.8% (1,110/1,341) (95% CI, 80.6 to 84.8%).

### Increased VT detection using the microarray.

**FIG 4:**
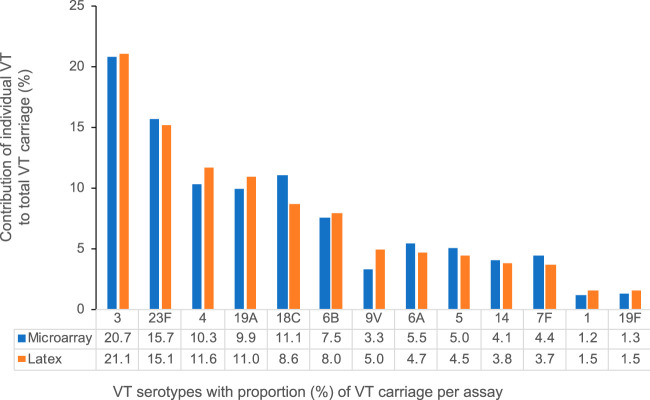
Proportions of individual vaccine serotypes contributing to total VT carriage. The proportions of individual VT detected by the microarray and by latex agglutination are not significantly different. Refer to Table S1 in the supplemental material for the reported frequency of each VT detected by the microarray and latex agglutination.

Using a larger study database of 1,949 samples from the same study, we evaluated latex agglutination and microarray data. When all ages (i.e., child and adult) were aggregated, there was an increase of 31.5% in VT prevalence by the microarray over that by latex serotyping: a 43.0% increase in VT carriage among children 3 to 6 years old, a 21.7% increase among children 5 to 10 years old, and a 10.8% increase among HIV-infected adults on ART (Table 1). This was due to the existence of samples reported as NVT by latex agglutination that also carried VT, as detected by the microarray. These VT, undetected by latex serotyping, were carried at lower relative abundances (median, 8%; range, 2% to 48%). The prevalence of multiple-serotype carriage (range, 2 to 6 serotypes) was 35.2% (686/1,949). Prevalences among the respective age groups were 44.4% (457/1,029), 32.8% (169/515), and 14.8% (60/405). Among samples with multiple-serotype carriage, latex agglutination identified the dominant serotype in 85.3% (585/686) (95% CI, 82.4 to 87.8%) of samples. Despite the overall increase in detection of VT carriage, the proportions of individual VT detected by the microarray and latex agglutination are not different (Fig. 4). See Table S1 in the supplemental material for the reported frequency of each VT detected by the microarray and latex agglutination.

**TABLE 1:** Increased detection of VT carriage by the microarray versus latex agglutination^*a*^

Group	Latex agglutination		Microarray		% increase in VT prevalence
	No. of samples	VT prevalence (%) (95% CI)	No. of samples	VT prevalence (%) (95% CI)	
Children					
3–6 yr old, PCV vaccinated (*n* = 1,360)	272	20.0 (17.9, 22.2)	389	28.6 (26.2, 31.1)	43.0
5–10 yr old, not PCV vaccinated (*n* = 904)	191	21.1 (18.5, 23.9)	240	26.5 (23.7, 29.6)	21.7
Adults, 18–40 yr old, HIV infected, not PCV vaccinated (*n* = 963)	137	14.2 (12.1, 16.6)	160	16.6 (14.3, 19.1)	10.8
Total (*n* = 3,227)	600	18.6 (17.3, 20.0)	789	24.4 (23.0, 26.0)	31.5

a VT, vaccine serotype; CI, confidence interval; PCV, pneumococcal conjugate vaccine.

### Key parameters of selected serotyping methods.

Table 2 presents key parameters to consider further in deciding which assay is appropriate for a particular setting. The estimated costs and feasibility of implementation and maintenance are specific to the setting in Malawi at the Malawi-Liverpool-Wellcome Trust Clinical Research Program in Blantyre. Extrapolation would need further validation outside the scope of this evaluation. Though more limited in its reporting of only a single serotype, latex agglutination is highly accurate, is less costly, and requires less expertise and fewer resources for field implementation and analysis. While the microarray is the costliest option, it provides greater accuracy with regard to total pneumococcal carriage, including multiple-serotype carriage and the relative abundances of individual serotypes carried. Whole-genome sequencing is a strong alternative to latex agglutination and would be nearly cost free if the sequence libraries were already available. In addition, WGS provides the opportunity for further analyses, including population structure and antibiotic resistance.

**TABLE 2:** Key comparative parameters of serotyping methods^*a*^

Parameter	Assessment		
	Latex agglutination (phenotypic)	Microarray (genomic)	PneumoCaT (genomic)
Assay implementation			
Sample used in assay	Pure growth from single isolate	Original sample in STGG	Pure growth from single isolate
Cost estimate^*b*^	Lowest of the three assays	Highest of the three assays	Middle of the three assays
Implementation of assay^*b*^	Least difficult (relatively simple)	Most difficult	Moderately difficult
Training required for implementation	Minimal	Advanced	DNA extraction: moderate WGS library manipulation: advanced PneumoCaT tool: moderate
Training required for processing and interpretation of results	Minimal	Moderate	Moderate
Assay output and interpretation			
Serotypes reported	Single	Multiple, if present	Single
NVT differentiation	No^*c*^	Yes	Yes
Relative abundance of individual serotypes reported	No	Yes	No
Additional outputs	Isolates archived and available for further analyses	AMR profile^*d*^ NT differentiation	WGS library accessible for further analyses, including population structure and AMR
Conclusion	Adequate for surveillance Limited resolution for optimal VE estimation	Cost and technique limits ability to decentralize implementation Detection of VT in low relative abundance is of critical importance Sentinel sites should be considered for regional NVT and VT resolution for optimal VE estimation	Limited resolution for optimal VE estimation No benefit over latex unless WGS library already available

a AMR, antimicrobial resistance; NT, nontypeable; VE, vaccine effectiveness.

b The estimated costs and feasibility of implementation and maintenance are specific to the study requirements and laboratory capacity (including no capacity for WGS or microarray) at the Malawi-Liverpool-Wellcome Trust Clinical Research Programme in Blantyre, Malawi.

c NVT and NT isolates are reported as NVT. The use of both commercial products and latex serotyping reagents produced in-house can significantly increase the number of NVT serotypes that can be differentiated by latex agglutination.

d An AMR profile cannot be assigned to a single strain in a sample with multiple-serotype or multiple-pathogen carriage.

## DISCUSSION

We report high concordance among three serotyping techniques applicable to routine pneumococcal surveillance. Importantly, we have extended the analysis to include relevant parameters beyond accuracy, including cost, time to result, and measures of input required for assay implementation and maintenance. These are parameters that researchers and policy makers should consider when deciding which assay to implement. All three assays appear accurate and concordant in identifying the dominant serotype.

While latex agglutination is accurate, requires the least expertise and resources for field implementation and analysis, and provides rapid results, standard latex approaches are not optimal for the surveillance of vaccine impact, including the detection of multiple-serotype carriage and of VT at low relative abundances (19). There have been attempts to implement latex agglutination for detection of multiple-serotype carriage. Gratten et al. serotyped as many as six colonies from nasal-swab culture plates and found multiple-serotype carriage in 29.5% of Papua New Guinean children (20). The authors went on to serotype at least 50 colonies from 10 selected nasal-swab cultures and concluded that the minor serotype carried accounted for 4% to 27% of the total pneumococcal population. A review of published data on multiple carriage concluded that it would be necessary to serotype at least five colonies in order to have a 95% chance of detecting a minor serotype if it accounted for 50% of the total pneumococcal population, and one would need to examine 299 colonies if the serotype was present at a relative abundance of 1%. As part of the PneuCarriage Project, to thoroughly characterize samples, as many as 120 colonies from each sample were selected to achieve >99% power to detect a minor serotype with an abundance of 5% (13). This approach would not be cost- or time-effective. Though dependent on technical capacity to develop in-house reagents, researchers in The Gambia developed a latex agglutination technique in which colonies from the primary culture plate are suspended in saline and serotyped by latex agglutination (21). While not differentiating NVT serotypes, they did show that up to 10.4% of pneumococcal acquisitions were found to be of multiple serotypes in a longitudinal infant cohort study. While latex serotyping is limited in its output, the process can be leveraged for additional endpoints, including, for example, measuring carriage density by counting CFU on agar culture plates. What is more, although this strategy is less cost- and time-effective, the use of both commercial products (including those from the Statens Serum Institute, Denmark) and latex serotyping reagents produced in-house has been well documented to significantly expand the number of NVT that can be differentiated by latex and to improve quality control procedures (11, 22).

With open-source bioinformatic tools such as PneumoCaT, serotyping by sequencing can be less costly than the microarray, even accounting for the costs of DNA extraction and WGS, while still being able to differentiate nontypeable isolates and nearly every known VT and NVT. Although we would not recommend initiating DNA extraction and WGS for the use of PneumoCaT alone, sequence libraries can be further leveraged for extensive informative bioinformatic analyses, useful in population biology, antimicrobial resistance investigations, and vaccine monitoring. Moreover, the use of PneumoCaT for serotyping would be essentially cost-free if the sequence libraries were already available, apart from the limited bioinformatic skills needed. While the microarray is more costly, it differentiates NVT and multiple-serotype carriage with relative abundance, as well as identifying non-S. pneumoniae contaminants (i.e., Streptococcus mitis, Streptococcus salivarius, and Staphylococcus aureus) with a degree of precision. This technique stands out for its sensitivity, in that it can detect serotypes at low relative abundances, which is of critical importance for understanding the transmission patterns of S. pneumoniae. Having the extra counts for each serotype from the same number of samples, as provided by the microarray, also has the advantage of adding power to a study’s statistics.

There are a number of limitations to mention, including the number of serotyping methods which were not evaluated, such as PCR and the SeroBA pipeline. SeroBA is a relatively new serotyping-by-sequencing software. With accuracy similar to that of PneumoCaT, SeroBA does have operational advantages (23). SeroBA can correctly call a serotype with a read coverage as low as 10× (20× is required for PneumoCaT). Using a k-mer-based approach, rather than the raw sequence alignment, SeroBA requires much lower computational power and time. On the other hand, the PneumoCaT source code can be easily adapted to operator needs, and both softwares are likely to run on a standard server configuration. Alternative culture-independent methods, such as isolation-independent conventional multiplex PCR-serotyping (cmPCR), could be important for confirming carriage when reculturing of original NP swab samples is not feasible. Although cmPCR has been successfully applied on DNA extracted directly from NPS-STGG, evidence suggests that cmPCR serotyping after culture enrichment returns a higher sensitivity and an ability to identify multiple-serotype carriage (9). Nonetheless, cmPCR can be confounded by nonpneumococcal streptococci (including S. mitis, Streptococcus oralis, and Streptococcus parasanguinis) (24). Due to high sequence similarity with target serotype-specific amplicons, cmPCR can overestimate pneumococcal carriage. Carvalho et al., for example, reported that 82.5% of samples (combined nasopharyngeal and oropharyngeal swabs) that were positive for pneumococci by cmPCR were culture negative by latex agglutination. Similarly, 35.0% of those positive for pneumococci by cmPCR were negative by *lytA*-pneumococcus-specific PCR. This is particularly problematic when bacterial DNA is extracted from culture-enriched naso- and oropharyngeal samples, requiring PCR results to be confirmed by latex agglutination or other serotyping procedures (24). Additional PCR limitations include the need for region-specific reaction protocols, implementing a high number of primer pairs to identify the same range of serotypes identified by the microarray or WGS, and the increased risk of detecting nonviable pneumococci. Since there is no evidence of a viable but nonculturable (VBNC) state in S. pneumoniae (25), identifying nonviable pneumococci could be disadvantageous for field-based research. While a formal economic analysis of the methods would be justified, we were unable to extrapolate the individual cost components between sites. Such components would include local salaries and additional labor costs, procurement and shipping of equipment and consumables, equipment maintenance, local health and safety requirements, and institutional costs. For this reason, comparative costing is grossly categorized. Although we did not include invasive isolates (from blood or cerebrospinal fluid, for example), it is important to identify serotypes associated with IPD, including in post-PCV impact studies. For invasive isolates, with a single-serotype sample expected, the microarray would have limited advantage. Application of serotyping by sequencing would then be the most informative option, including insight into population structure, antimicrobial resistance patterns, and serotype replacement disease.

### Conclusion.

Selection of the appropriate assay should be based on the intended analysis and endpoint. While accuracy and concordance are high between the three assays, parameters of field implementation and cost vary significantly. In a setting of limited resources, as is true throughout much of sub-Saharan Africa, latex agglutination is the best overall option for decentralized surveillance of vaccine impact. However, WGS, which adds population structure, and the microarray, which adds multiple-serotype carriage, should be considered at regional reference laboratories for investigating the importance of VT at low relative abundances in transmission and disease.

## Supplementary Material

Supplemental file 1
