# Supplementary material for: Evaluation of Pneumococcal Serotyping of Nasopharyngeal-Carriage Isolates by Latex Agglutination, Whole-Genome Sequencing (PneumoCaT), and DNA Microarray in a High-Pneumococcal-Carriage-Prevalence Population in Malawi
Source: J Clin Microbiol. 2020 Dec 17;59(1):e02103-20. doi: 10.1128/JCM.02103-20 (PMC7771446; doi:10.1128/JCM.02103-20)
Supplement: Supplemental file 1 [file JCM.02103-20-s0001.pdf]

Supplemental Material

Swarthout T.D., Gori A., et al.

Text S1: Description of each assay

Serotyping by latex agglutination: This is performed on pure growth cultures of pneumococci. It is an agglutination method using anti-rabbit IgG-coated latex particles sensitized to pooled and select individual pneumococcal serotype-specific antisera (PCV13 serotypes: 1, 3, 4, 5 6A, 6B, 7F, 9V, 14, 18C, 19A, 19F, 23F) for serogrouping and serotyping *S. pneumoniae*. Visible agglutination occurs when the *S. pneumoniae* capsular antigen reacts with the antibody-coated latex beads. Commercially available kits, including the Pneumotest-Latex^{TM} kit, are intended for partial typing of pure cultures of pneumococci, differentiating vaccine serotypes with both non-typeable and NVT reported as NVT.

Molecular serotyping by microarray. This assay is performed on DNA extracts of original NP swab samples using the B_G@S SP-CPS v1.4.0 microarray, designed for *S. pneumoniae*. Serotyping by microarray utilises the DNA extracted from a primary culture plate, where an aliquot of the original NP swab storage medium is grown. The main strength of this technique is to differentiate and report nearly all known serotypes, including relative abundance of each in the case of multiple serotype carriage (MSC). Other outputs, though less robust, include presence of other bacterial species and genetic characteristics, including antimicrobial resistance (AMR) and genome-group based on presence and absence of core genes in the original sample. Characteristics such as AMR are less robust, for example, because the AMR profile cannot be assigned to a single strain in a sample with multiple-serotype or multiple-pathogen carriage. Final microarray results are accessible from Bugs Bioscience's password-protected SentiNet website as a Microsoft Excel spreadsheet or a tab-separated text file. As with PneumoCaT, described below, microarray outputs are generally accessed, with limited guidelines for use or interpretation. Though less formidable than PneumoCaT, this requires some further reading and discussion with the Bugs Bioscience's team. Examples of terminology include '-like' (e.g. 23F-like) and including an '*' (asterisk) with some reported serotypes. Some closely related serotypes are

reported as a group, with the individual serotype call in brackets (e.g., 6A/B [6B]). The Bugs Bioscience produces a very useful web-based dashboard of pre-programmed analyses.
Note: For the purpose of this analysis, identified serotypes marked with an '*' (asterisk) were removed.

Serotyping-by-sequencing using PneumoCaT. The rapid reduction in the cost of whole genome sequencing (WGS) has led to its extensive use in the monitoring of pneumococcal serotypes. Developed in 2015 by Public Health England (PHE), PneumoCaT (**Pneumo**coccal **Ca**psule **T**yping) offers a fully functional automated pipeline for serotyping *S. pneumoniae* WGS data.[1] As an opensource bioinformatic tool, PneumoCaT was developed to meet the needs of the initial owner, in this case PHE. The main drive for PHE's developer was the serotyping of invasive pneumococcal isolates, where MSC is rare and a limited variability of serotypes is recorded. As such, in studies regarding invasive pneumococcal disease (IPD), PneumoCaT was initially reported to identify over 90% of pneumococcal serotypes but the database is regularly corrected and updated.[2]

Although the PneumoCaT tool includes all the steps to determine serotype from raw (unassembled) DNA sequences, the user is required to perform a number of steps to obtain those sequences, including DNA extraction from bacterial isolates and DNA sequencing (in-house or externally). Bioinformatic and IT competencies will be needed if working with raw WGS and the user is not familiar with the command-line environment. The user will need to review the raw WGS data files for contamination and cleaning of low-quality DNA segments in the sequence libraries. If the user has WGS libraries available (e.g. for other study objectives), then there are no additional material costs. If DNA extraction and sequencing is required, costings increase accordingly. As a tool designed by scientists for scientists, the interface can be challenging to those not familiar with the format for inputting sequence libraries and interpreting output. As with Microarray, PneumoCaT is generally accessed by researchers with relevant training, with limited guidelines for use or interpretation of outputs. This requires time in reviewing the relevant websites, publications or willingness to contact the tool owner directly.

REFERENCES

1. Kapitai 2016; Kapatai G, et al. Whole genome sequencing of Streptococcus pneumoniae: development, evaluation and verification of targets for serogroup and serotype prediction using an automated pipeline. PeerJ **2016**; 4:e2477

2. https://github.com/phe-bioinformatics/PneumoCaT

Table S1: Frequency of vaccine serotype (VT) detected by microarray and latex, as reported in Figure 4 in the manuscript. Having the extra counts for each serotype from the same number of samples, as provided by microarray, has the advantage of adding power to a study's statistics.

Serotype	Microarray (%)	N	Latex (%)	N	% increase
3	20.7	174	21.1	127	37.0%
23F	15.7	86	15.1	70	22.9%
4	10.3	34	11.6	23	47.8%
19A	9.9	63	11.0	48	31.3%
18C	11.1	37	8.6	22	68.2%
6B	7.5	42	8.0	27	55.6%
9V	3.3	46	5.0	28	64.3%
6A	5.5	83	4.7	66	25.8%
5	5.0	10	4.5	9	11.1%
14	4.1	93	3.8	52	78.8%
7F	4.4	11	3.7	9	22.2%
1	1.2	28	1.5	30	-6.7%
19F	1.3	132	1.5	91	45.1%
**Total**		**839**		**602**	

[1] Percent increase: ([frequency of serotype by microarray - frequency of serotype by latex] / frequency of serotype by latex)*100%
